# Probiotic-Derived Bioactive Compounds in Colorectal Cancer Treatment

**DOI:** 10.3390/microorganisms11081898

**Published:** 2023-07-27

**Authors:** Christina Thoda, Maria Touraki

**Affiliations:** Laboratory of General Biology, Department of Genetics, Development and Molecular Biology, School of Biology, Faculty of Sciences, Aristotle University of Thessaloniki, 54 124 Thessaloniki, Greece; christhoda@bio.auth.gr

**Keywords:** probiotics, gut microbiota, bioactive compounds, colorectal cancer

## Abstract

Colorectal cancer (CRC) is a multifactorial disease with increased morbidity and mortality rates globally. Despite advanced chemotherapeutic approaches for the treatment of CRC, low survival rates due to the regular occurrence of drug resistance and deleterious side effects render the need for alternative anticancer agents imperative. Accumulating evidence supports that gut microbiota imbalance precedes the establishment of carcinogenesis, subsequently contributing to cancer progression and response to anticancer therapy. Manipulation of the gut microbiota composition via the administration of probiotic-derived bioactive compounds has gradually attained the interest of scientific communities as a novel therapeutic strategy for CRC. These compounds encompass miscellaneous metabolic secreted products of probiotics, including bacteriocins, short-chain fatty acids (SCFAs), lactate, exopolysaccharides (EPSs), biosurfactants, and bacterial peptides, with profound anti-inflammatory and antiproliferative properties. This review provides a classification of postbiotic types and a comprehensive summary of the current state of research on their biological role against CRC. It also describes how their intricate interaction with the gut microbiota regulates the proper function of the intestinal barrier, thus eliminating gut dysbiosis and CRC development. Finally, it discusses the future perspectives in precision-medicine approaches as well as the challenges of their synthesis and optimization of administration in clinical studies.

## 1. Introduction

Colorectal cancer (CRC) represents the third most prevalent form of cancer worldwide, accounting for more than 1.9 million new incidents and 900,000 deaths in 2020 [1,2]. CRC is a multifactorial and heterogeneous non-communicable disease. Approximately 65% of CRC cases develop sporadically through the accumulation of acquired somatic mutations and epigenetic modifications [3], while other cases are associated with CRC predisposition genes (25%) and hereditary syndromes (5%) [4,5]. The development of sporadic CRCs is usually a multistep and long-lasting process that involves progressive transformation of normal intestinal epithelial cells (IECs) into malignant cells [2]. Based on the epithelial lesion type and the specific molecular profile, two distinct carcinogenesis pathways have been recognized [1]: the traditional adenoma–carcinoma pathway [6] and the serrated neoplasia pathway [7].

Expedient options of CRC treatment, including surgery, radiotherapy, targeted therapy, immunotherapy, and chemotherapy [1], are based on tumor-related features [8]. Chemotherapeutic intervention with palliative purposes is often selected to enhance the survival rate of CRC patients [3]. However, the efficacy of current strategies is usually imperiled, due to drugs’ inadequate capacity to discriminate between healthy and cancer cells, thus causing intensified toxicity and undesirable effects to the human body [2]. Additionally, chemotherapy rarely accomplishes the complete eradication of malignant cells, while it can promote drug-resistance development [9]. Cancer cells display a variety of remarkable changes in their physiology [10]. Recently, this list has been expanded to incorporate the effects of gut microbiota composition on the initiation and/or progression of tumorigenesis [11].

Gut microbiota, the complex microbial population inhabiting the gastrointestinal tract (GIT), can shape oncologic outcomes in several ways [12]. Global epidemiological studies suggest that variations in gut microbiota composition and diversity, known as gut dysbiosis, can influence CRC initiation and progression [13]. The involvement of gut microbes in modulating physiological processes could be reversed in case of dysbiosis towards chronic inflammation and CRC induction [14]. Depending on the predominant gut microbiota-induced fermentation pathway, dietary patterns reflect on the enrichment of oncometabolites or tumor-suppressing metabolites [15]. Gut microbiota metabolism is closely associated with the efficacy and toxicity of traditional chemotherapeutic treatments [16]. In some cases, gut metabolites induce an immunostimulatory tumor microenvironment (TME) that advocates drugs toxicity on tumor cells [17]. However, a substantial number of patients experience treatment-associated adverse effects or even mortality due to these medications, a fact attributed to their intestinal microbial diversity [16]. Deciphering the synergistic or contradictory action of gut microbiota with anticancer agents remains a conundrum.

**Figure 1:**
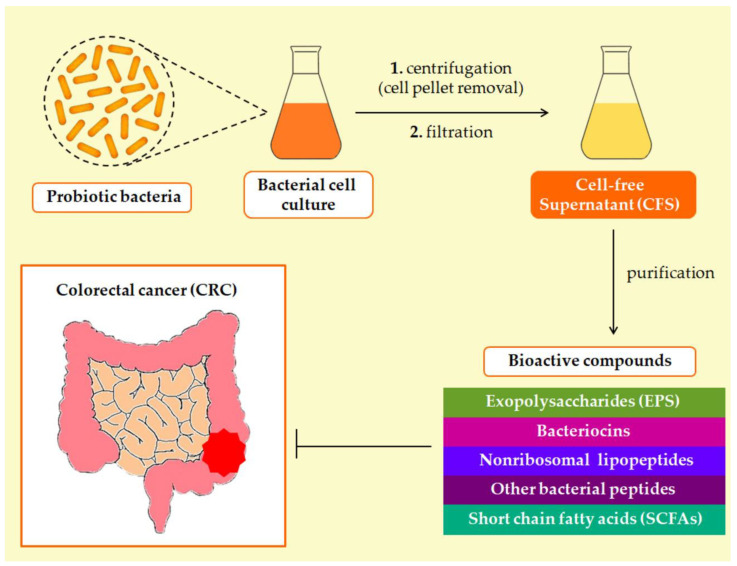
Diagrammatic representation of probiotic-derived bioactive compounds with antiproliferative properties against CRC.

In recent years, probiotics have been utilized to attenuate postoperative gastrointestinal complications in eligible CRC patients undergoing chemotherapy [18]. Lactic acid bacteria (LAB), a ubiquitous group of Gram-positive microorganisms, comprise the most common type of probiotics due to their beneficial health effects on the host and their “generally recognized as safe” (GRAS) status [19]. The anticancer activity of probiotics is predicated on their ability to suppress tumor growth as well as induce cell cycle arrest and apoptosis [20,21]. Nevertheless, probiotics have been reported to biotransform chemical compounds [22] and anticancer agents [23], thus affecting drugs’ bioavailability and therapeutic outcomes, or even leading to disease exacerbation [24]. In this regard, administration of probiotic-derived bioactive compounds with profound anti-inflammatory and antiproliferative properties is now being leveraged as a novel personalized therapeutic approach in CRC treatment [25]. These secreted metabolic products purified from the probiotic cell-free supernatant (CFS) comprehend short-chain fatty acids (SCFAs), bacteriocins, exopolysaccharides (EPSs), nonribosomal lipopeptides, and other bacterial peptides [26] (Figure 1).

This review provides a concise summary of the most prominent probiotic-derived compounds and emphasizes the current knowledge regarding their biological roles in CRC treatment. Additionally, it focuses on the underlying mechanisms that configure their antiproliferative effects on malignant cells, as well as how their intricate interaction with the gut microbiota can lead to the elimination of dysbiosis. Finally, it discusses the future perspectives and challenges of their administration to individuals.

## 2. The Role of Gut Microbiota in CRC Development and Treatment

The human gut microbiota encompasses about a hundred trillion microorganisms, a number three times greater than the total number of human cells [5,27], while it encodes more than three million genes [28]. This diverse microbial community harbors between 500 to 1000 bacterial species, mostly belonging to the Bacteroidetes and Firmicutes phyla [29], and is strictly compartmentalized to the intestinal lumen of the GIT. The establishment of the gut microbiota occurs during infancy [30], while it stabilizes and reaches its peak complexity in adulthood [31]. Its composition varies among individuals and is predominantly shaped by various factors including nutrition [32], antibiotic treatment [33], age, and ethnicity [34]. A healthy gut microbiota plays a fundamental role in host nutrient metabolism, [35], immunomodulation [36,37], maintenance of the mucosal barrier integrity [38], production of antimicrobial and anti-inflammatory compounds [39,40], and protection against intruding pathogens [5].

The qualitative and quantitative alterations in the gut microbiota composition, namely dysbiosis, are often associated with increased susceptibility to gastrointestinal malignancies [41]. Gut dysbiosis is characterized by the overgrowth of proinflammatory bacterial species at the expense of beneficial microbes [42], thus leading to disturbance of epithelial barrier function, chronic inflammation, oxidative stress [43], and colorectal tumorigenesis [44]. In fact, approximately 20% of cancers, including CRC, are hypothesized to be attributed to microbes [45]. Several studies have indicated the association between gut microbiota dysbiosis and cancer development [46,47,48,49]. CRC occurrence is generally associated with the prevalence of specific bacterial species [50,51], such as *Fusobacterium nucleatum* [52], *Bacteroides fragilis* [53,54], *Escherichia coli* [55], *Streptococcus bovis* and *Streptococcus gallolyticus* [56], *Helicobacter pylori* [57], *Salmonella typhimurium* [58], *Clostridium* sp. [59], and *Citrobacter rodentium* [60]. These microorganisms induce carcinogenesis via activation of signaling pathways, toxic metabolites production, and extensive DNA damage [41].

The role of the gut microbiota in CRC development is supported by multiple studies [61,62,63,64,65,66] and explained by the “Driver-Passenger” model [67]. According to this model, “driver” pathogenic bacteria can induce colorectal tumorigenesis via the production of toxins and digestion of the protective mucus layer shielding the intestinal epithelium. The CRC microenvironment stimulates the overgrowth of specific “passenger” opportunistic bacteria that further promote dysbiosis, epithelial cell hyperproliferation, and chronic inflammation, thus leading to CRC progression [68]. In vivo studies confirmed that fecal microbiota transplantation (FMT) from CRC patients into germ-free [69] and Adenomatous polyposis coli (APC) gene knockout mice [70] facilitated intestinal tumorigenesis. Furthermore, significant differences regarding gut microbiota composition were detected between healthy individuals and CRC patients, which were correlated with the expression of genes known to promote inflammatory responses [71,72]. The gut microbiota may be harnessed through establishing microbial therapeutics as chemopreventive agents [73,74,75,76,77,78,79,80], as adjuvants to augment drug efficacy [81,82], or as diagnostic biomarkers for CRC screening [83,84,85,86,87].

Several studies have indicated that medications can significantly affect the gut microbiota, thus playing a pivotal role in disease development and therapy [85,88,89]. At the same time, gut microbes are implicated in drug pharmacokinetics [90], pinpointing this as one of the most challenging aspects of developing individual-specific anticancer agents to improve therapeutic outcomes. Surprisingly, the role of the gut microbiota in CRC therapy is rather supported by conflicting results. The contribution of the gut microbiota in enhanced therapeutic efficacy has been reported, while, concomitantly, the metabolic activity of gut bacteria is a critical trait in side effect exacerbation. For instance, irinotecan is mostly used intravenously to treat CRC. However, it commonly causes severe diarrhea upon its conversion into the active metabolic form by microbial β-glucuronidase enzymes. Those side effects are mitigated via the utilization of β-glucuronidase inhibitors [90]. Additionally, 5-Fluorouracil (5-FU) was found to inhibit the growth of CRC-related *F. nucleatum*, while specific intratumoral microbiota members can covert 5-FU into a nontoxic form, thus resulting in increased cancer epithelial cell growth [91].

## 3. Probiotic Derived Bioactive Compounds and CRC

### 3.1. Cell-Free Supernatant

The cell-free supernatant (CFS) encompasses diverse bioactive metabolites secreted by probiotics during microbial growth [25,92] as well as the remains of the culture medium [93]. The typical procedure for CFS preparation involves two main steps: the removal of bacterial cells via centrifugation and the filtration of the emerged mixture to obtain a sterile, transparent liquid medium [94,95] (Figure 1). Optionally, the CFS can be subjected to lyophilization prior to use [96]. The compositional profile of CFS-derived compounds, ranging from proteinaceous molecules to organic acids, is generally altered by individual nutrients in the growth medium [96], thus endowing the CFS with variegated health-promoting effects [92,93].

The antiproliferative properties of CFSs against CRC cells originate from probiotics, mainly of the genus *Lactobacillus* [97,98,99,100,101,102,103,104,105,106,107], *Bacillus* [108,109], *Enterococcus* [110], *Bifidobacterium* [111], *Leuconostoc* [112], or commensal bacteria [113,114] and have been accredited in vitro (Table 1). The CFS can promote the activation of the intrinsic apoptotic pathway as indicated by increased caspase expression [115,116,117,118,119,120] and other major biochemical changes, including the loss of mitochondrial membrane potential and cytochrome c release, down-regulation of the anti-apoptotic *BCL2* gene, and the up-regulation of the pro-apoptotic *BAK*, *BAD*, and *BAX* genes [121,122]. Moreover, apoptosis-associated morphological alterations such as the formation of cytoplasmic blebs, chromatin condensation, and DNA fragmentation have been observed [123,124,125,126]. In addition, the potent anti-inflammatory properties of *Pediococcus acidilactici* supernatant in LPS-pretreated CRC cells [127], as well as the suppression of pro-inflammatory cytokine production in pathogen-stimulated Caco-2 cells by the CFS from *Lacticaseibacillus* sp. [128] have been reported. Interestingly, *Clostridium butyricum* TO-A supernatant significantly down-regulates Toll-like receptor-4 (TLR4) expression and this effect is attributed to the high content of butyrate [129].

The anti-metastatic effects of various CFS have been previously observed [130,131,132,133,134,135]. For instance, CFS derived from *Lactobacillus rhamnosus* GG was found to prevent CRC cell invasion via reduction of matrix metalloproteinase-9 (MMP-9) expression and increased tight junction protein zona occludens-1 (ZO-1) and tissue inhibitor of metalloproteinase (TIMP) levels [130,131], whereas it exhibits synergistic action with 5-FU [136]. *Lactiplantibacillus plantarum* and *Lactobacillus fermentum* CFS induced a dramatic increase in apoptosis marker levels in three-dimensional (3D) spheroids of CRC cells in vitro [137,138]. In addition, various CFSs have been reported to suppress the expression of cyclin genes, thus affecting cell cycle progression [139,140]. A CFS derived from *Lactobacillus plantarum* CCARM 0067 resulted in Wnt/β-catenin suppression when combined with 5-FU [141], while it contributed to the restoration of sodium-coupled monocarboxylate transporter 1 (SMCT1) expression leading to butyrate-induced antiproliferative effects in 5-FU resistant CRC cells [142]. The inhibition of autophagy-related proteins and synergistic effects with chloroquine were observed in the case of *Lactobacillus plantarum* CFS-treated CRC cells [143]. Lastly, there is also evidence of the beneficial effects of CFSs derived from yeast cultures [144,145].

**Table 1:** In vitro effects of cell-free supernatants on CRC cells.

Probiotic Strain	CRC Cell Line	Effect/Mechanism of Action	Reference
*Bacillus coagulans* Unique IS2	COLO 205	cytotoxic effect, apoptosis induction (↑ Bax/Bcl-2 ratio/ MtMP loss/cyt c release/↑ caspase-3/PARP cleavage)	[116]
*Bacillus polyfermenticus*	HT-29, DLD-1, Caco-2	antiproliferative activity, ErbB-2 and ErbB-3 inhibition	[108]
*Bacillus polyfermenticus* KU3	LoVo, HT-29	anti-inflammatory and cytotoxic activity	[109]
*Bifidobacterium adolescentis* SPM0212	HT-29, SW-480, Caco-2	dose-dependent anticancer activity, changes in cellular morphology, ↓ TNF-α, inhibition of harmful fecal enzymes	[124]
*Bifidobacterium bifidum*	SW742	cytotoxic effect	[111]
*Clostridium butyricum* TO-A	HT-29	TLR4 down-regulation	[129]
*Enterococcus faecium* 12a *E. faecium* L12b *E. hirae* 20c	HCT-15	dose-dependent cytotoxic effect, apoptosis-related morphological changes	[125]
*E. lactis* IW5	HT-29, Caco-2	time- and dose-dependent cytotoxic activity, extrinsic apoptotic pathway	[110]
*Faecalibacterium prausnitzii*	HCT 116	time- and dose-dependent cytotoxic activity	[113]
*Lacticaseibacillus paracasei* SD1, *Lacticaseibacillus rhamnosus* SD4, SD11 and GG	Caco-2	dose-dependent cytotoxic effect, pro-inflammatory cytokine suppression after stimulation with pathogens	[128]
*Lactiplantibacillus plantarum* 0991	Caco-2	dose-dependent antiproliferative activity, ↑ oxidative stress, intrinsic apoptotic pathway	[120]
*Lactiplantibacillus plantarum* L125	HT-29	antiproliferative, anti-clonogenic and anti-migration activity	[135]
*Lactiplantibacillus plantarum* OC01	HCT 116, HT-29	dose-dependent cell toxicity (2D/3D-spheroid cultures), mTOR and ERK pathways suppression, E- to N-Cadherin switch inhibition	[138]
*Levilactobacillus brevis* 0983	Caco-2	dose-dependent antiproliferative activity, ↑ oxidative stress, intrinsic apoptotic pathway	[120]
*Lactobacillus* spp.			
*L. acidophilus* ATCC 43121	HT-29	antiproliferative and antioxidant properties, apoptosis induction (↑ caspase-3,-9/↑ Bax/Bcl-2 ratio)	[121]
*L. acidophilus* CICC 6074	HT-29	time- and dose-dependent cytotoxic activity, cell cycle arrest (G0/G1), intrinsic apoptotic pathway (MtMP loss/cyt c release/↑ *BAX*, *CASP3*, *CASP9*/↓ *BCL2*)	[117]
*L. acidophilus* IIA-2B4	WiDr	dose-dependent anticancer activity	[106]
*L. brevis* PM177	HT-29	dose-dependent cytotoxic effect	[101]
*L. casei* ATCC 334	HCT 116	anti-metastatic effects (↓ MMP-9/↑ ZO-1)	[130]
*L. casei* ATCC 393	HT-29	antiproliferative effect	[100]
*L. casei* M3	HT-29, Caco-2	antiproliferative and anti-migration activity, VEGF/MMPs signaling pathway down-regulation	[134]
*L. casei* strains	Caco-2	dose-dependent cytotoxic effects, apoptosis induction	[107]
*L. crispatus* SJ-3C-US	HT-29	anti-metastatic effects (↓ *MMP2* and *MMP9*/ ↑ *TIMP1* and *TIMP2*)	[131]
*L. delbrueckii*	SW-620	dose-dependent anticancer activity, anti-metastatic effects, cell cycle arrest (G1), intrinsic apoptotic pathway	[115]
*L. delbrueckii* ATCC 11842	HT-29	antiproliferative and antioxidant properties, apoptosis induction (↑ caspase-3,-9/↑ Bax/Bcl-2 ratio)	[121]
*L. fermentum*	DLD-1, HT-29, WiDr	dose-dependent cytotoxic activity (2D/3D-spheroid cultures), apoptosis markers, NF-κB pathway inhibition	[137]
*L. fermentum* NCIMB 5221	SW-480, Caco-2	time-dependent antiproliferative effect, apoptosis induction	[98]
*L. johnsonii* LC1	HT-29, HT29-dx	↓ cell viability, ↑ mitochondrial ROS production	[103]
*L. pentosus* S3			
*L. pentosus* B281	Caco-2, HT-29	↓ cell proliferation, cell cycle arrest (G1), ↓ cyclin genes	[139]
*L. plantarum* A7	Caco-2, HT-29	antiproliferative effect	[97]
*L. plantarum* ATCC 14,917	Caco-2	time- and dose-dependent cytotoxic activity, intrinsic apoptotic pathway (↓ *BCL2*/ ↑ caspase-3, -9, *BAK*, *BAD*, and *BAX*)	[122]
*L. plantarum* B282	Caco-2, HT-29	↓ cell proliferation, cell cycle arrest (G1), ↓ cyclin genes	[139]
*L. plantarum* CCARM 0067	HT-29/5-FUR, HCT 116/5-FUR	↓ CSCs markers, caspase-3 dependent apoptosis and Wnt/β-catenin suppression in combination with 5-FU	[141]
	HCT 116/5-FUR	anti-metastatic effects, ↓ CLDN-1	[132]
	HCT 116, HCT 116/5-FUR	restoration of SMCT1 expression leading to butyrate-induced antiproliferative effect and apoptosis	[142]
*L. plantarum* IIA-1A5	WiDr	dose-dependent anticancer activity	[106]
*L. plantarum* KCTC 3108	Caco-2	↓ cell viability, ↓ autophagy-related proteins, induction of mitochondrial dysfunction, synergistic effect with chloroquine	[143]
*L. plantarum* S2 and O2	HT-29, HT29-dx	↓ cell viability, ↑ mitochondrial ROS production	[103]
*L. plantarum* strains	HT-29	antiproliferative effect, induction of apoptosis	[102]
*L. plantarum* YYC-3	HT-29, Caco-2	antiproliferative and anti-migration activity, VEGF/MMPs signaling pathway down-regulation	[134]
*L. reuteri* BCRC14625	HT-29	cell membrane damage, LDH release, Bcl-2 inhibition via ↑ NO production	[101]
*L. reuterii* DSM 17938	HT-29, HT29-dx	↓ cell viability, ↑ mitochondrial ROS production	[103]
*L. reuteri* NCIMB 701359	SW-480, Caco-2	apoptotic and antiproliferative activity	[99]
*L. reuteri* PTCC 1655	HT29-ShE	anti-metastatic properties, apoptosis induction, ↓ MMP-9 and COX-2, ↑ TIMP-1	[133]
*L. rhamnosus* ATCC 7469	Caco-2	time- and dose-dependent cytotoxic activity, intrinsic apoptotic pathway (↓ *BCL2*/ ↑ caspase-3, -9, *BAK*, *BAD*, and *BAX*)	[132]
*L. rhamnosus* GG	HCT 116	anti-metastatic effects (↓ MMP-9/↑ ZO-1)	[130]
	HT-29	anti-metastatic effects (↓ *MMP2* and *MMP9*/ ↑ *TIMP1* and *TIMP2*)	[131]
	HT-29, Caco-2	antiproliferative and anti-migration activity, VEGF/MMPs signaling pathway down-regulation	[134]
	HT-29, HT29-dx	↓ cell viability, ↑ mitochondrial ROS production	[103]
	HCT 116, Caco-2, HT-29	dose-dependent antiproliferative activity, mitotic arrest, synergistic action with 5-FU	[136]
*L. rhamnosus* MD 14	Caco-2, HT-29	antigenotoxic and cytotoxic activity, cell cycle arrest (G0/G1)	[105]
*L. rhamnosus* Y5	HT-29	time- and dose-dependent cytotoxic effect, cell cycle arrest (G0/G1), ↓ *CCND1*, *CCNE1* and *ERBB2*, apoptosis induction (↑ *CASP3*, *CASP9* and *BAX*/↓ *BCL2*)	[118]
*L. salivarius* Ren	HT-29	antiproliferative activity, apoptosis induction, AKT pathway inhibition, cyclin D1 and COX-2 suppression	[140]
*Lactobacillus* spp.	HT-29, Caco-2	cytotoxic activity, ↓ *ERBB2* and *ERBB3*	[104]
*Lactobacillus* spp.	HT-29	dose-dependent antiproliferative activity, irregular morphology and cell condensation, ↑ caspase-3,-8 and Bax	[119]
*Leuconostoc pseudomesenteroides* strains	Caco-2, HT-29	antioxidant and anticancer properties	[112]
*Pediococcus acidilactici* TMAB26	HT-29, Caco-2	cytotoxic effects, anti-inflammatory properties in LPS-pretreated cells (↓ TNF-α, IL-6/↑ IL-10)	[127]
Propionibacterium acidipropionici Propionibacterium freudenreichii	HT-29, Caco-2	cytotoxic activity, induction of apoptosis (MtMP loss/↑ ROS/↑ caspase-3/chromatin condensation)	[123]
*Propionibacterium freudenreichii* DSM 2027	HCT 116	dose-dependent cytotoxic activity at 72 h	[114]
*Steptococcus salivarius* CP163 *Streptococcus salivarius* CP208	HT-29	antiproliferative activity, apoptosis induction (↑ caspase-2, DNA fragmentation)	[126]
Yeasts			
*Kluyveromyces marxianus* PCH397	SW-480	cytotoxic and antioxidant properties, cell cycle arrest	[145]
*Pichia kudriavzevii* AS-12	HT-29, Caco-2	antiproliferative effect, apoptosis-related morphological changes, apoptotis induction (↑ *BAD*, *CASP3*, *CASP8*, *CASP9* and *Fas*/↓ *BCL2*)	[144]

↑: increase or up-regulation, ↓: decrease or down-regulation. Colon cancer cell lines: COLO 205, HT-29, DLD-1, Caco-2, LoVo, SW-480, SW742, HCT-15, HCT 116, WiDr, SW-620, HT29-dx (doxorubicin-resistant HT-29 cells), HT-29/5-FUR (5-Fluorouracil-resistant HT-29 cells), HCT 116/5-FUR (5-Fluorouracil-resistant HCT 116 cells), HT29-ShE: E-cadherin shRNA engineered HT-29. MtMP: Mitochondrial membrane potential, cyt c: cytochrome c, PARP: Poly (ADP-ribose) polymerase, TNF-α: Tumor necrosis factor-α, TLR4: Toll-like receptor 4, mTOR: mammalian target of rapamycin, *CASP*: caspase gene, MMP-9: Matrix metalloproteinase-9, ZO-1: Zonula occludens-1 protein, VEGF: Vascular endothelial growth factor, MMPs: Matrix metalloproteinases, *MMP*: Matrix metalloproteinase gene, *TIMP*: Tissue inhibitor of metalloproteinase gene, NF-κB: Nuclear factor-κB, ROS: Reactive oxygen species, CSCs: Cancer stem cells, 5-FU: 5-Fluorouracil, CLDN-1: Claudin-1, SMCT1: Sodium-coupled monocarboxylate transporter 1, LDH: Lactate dehydrogenase, NO: Nitric oxide, COX-2: Cyclooxygenase-2, *CCND1*: cyclin D1 gene, *CCNE1*: cyclin E1 gene, *ERBB2*: ErbB-2 receptor tyrosine kinase 2 gene, LPS: Lipopolysaccharide, IL-6: Interleukin-6, IL-10: Interleukin-10, *Fas*: Fas cell surface death receptor gene.

### 3.2. Exopolysaccharides

Exopolysaccharides (EPSs) have gained scientific interest in recent years due to their diverse health-promoting properties [146], including the inhibition of pathogens’ adhesion to the intestinal epithelium, the enhancement of gut barrier integrity, and the regulation of mucosal immune responses [147]. Bacterial EPSs are extracellular, long-chain, high-molecular-weight polysaccharides, distinguished by their complex structures, which are strain-dependent and attributed to their distinct functions [92,96]. EPSs could be structurally divided into homopolysaccharides (HoPSs) containing a single type of monosaccharide and heteropolysaccharides (HePSs) composed of repeating units of numerous monosaccharides [25,148].

Their anticancer activity has been extensively studied [149,150,151], especially in the case of *Lactobacillus*-retrieved EPSs [152]. The majority of studies have designated the dose- or/and time-dependent cytotoxic effect of EPSs on CRC cell lines in vitro (Table 2) [153,154,155,156,157,158,159,160]. In several cases, EPSs trigger the intrinsic apoptotic pathway activation, indicated by the increased expression of Bax, caspase-3, and caspase-9 [161,162] and decreased levels of Bcl-2 [163,164,165]. EPSs from *L. plantarum* NCU116 activate the c-Jun dependent Fas/FasL-mediated apoptotic pathway through TLR2 in mouse intestinal epithelial cancer cells [166]. Additionally, apoptosis induction in EPS-treated CRC cells was confirmed by distinct apoptosis-related morphological features, such as cell shrinkage, nuclear fragmentation, and chromatin condensation [167,168,169,170]. EPSs from *Lactobacillus acidophilus* 10307 can inhibit the expression of genes involved in tumor angiogenesis and survival, including vascular endothelial growth factor (VEGF) and hypoxia-inducible factor-1α (HIF-1α), while they up-regulate antiangiogenic gene expression, such as tissue inhibitor of metalloproteinase-3 (TIMP-3) [171]. They can also enhance peroxisome proliferator-activated receptor-γ (PPAR-γ) expression, thus contributing to the suppression of CRC cellular growth [172]. Interestingly, cell-bound EPSs (cb-EPSs) isolated from *L. acidophilus* 606 were found to promote cell death via autophagy in HT-29 cells [173], while EPSs from *Lactobacillus casei* 01 can repair 4-nitroquinoline 1-oxide (4-NQO)-damaged IECs [174]. Probiotic yeast-derived EPSs can hinder the AKT-1, mammalian target of rapamycin (mTOR), and JAK-1 pathways to induce apoptosis in several CRC cell lines [175].

### 3.3. Bacteriocins

Bacteriocins encompass a heterogeneous group of extracellular, ribosomally synthesized antimicrobial peptides (AMPs) [176]. Harnessing their multifaceted functions, including the elimination of CRC-associated bacterial pathogens while avoiding disruption of the commensal microbiota [25,177], the regulation of the host’s immune responses contributing to gut homeostasis [37], and cancer-cell-specific targeting ability [178], bacteriocins possess unique features as potential anticancer agents [179,180]. Salivaricin was found to display potent antimicrobial activity against *F. nucleatum* in an ex vivo model of the human colon, thus reducing CRC development risk [181]. Interestingly, a recent in vitro study divulged that bacteriocins can migrate across epithelial monolayers [182], supporting their ability to disseminate across the GIT to exert their beneficial effects [183].

The selective cytotoxicity of bacteriocins against cancer cells is rather attributed to three dominant dissimilarities between cancer and normal cells. Firstly, the negatively charged plasma membrane of cancer cells, due to anionic compound overexpression, facilitates the electrostatic interactions of cationic bacteriocins with higher affinity to cancer than normal cells [178]. With regard to the fact that premalignant cells undergoing transformation into metastatic CRC forms are characterized by changes in phospholipid content [184], bacteriocins could be utilized as selective cytotoxic agents without affecting healthy cells. For instance, duramycin decreases CRC cells proliferation through binding to phosphatidylethanolamine (PE) [185]. Additionally, high membrane fluidity, a feature known to confer metastatic capability to malignant cells, enables bacteriocins to debilitate cancer cells’ membrane stability [186]. Lastly, the existence of abundant microvilli on the cancer cell surface allows a greater amount of bacteriocins to penetrate tumor cells [187].

Nisin is the prominent lantibiotic produced by *Lactococcus lactis* subsp. *lactis*, existing in four natural variants (A, Z, Q, and F), which differ in one or two amino acids [188]. Nisin variants A and Z have been extensively examined for their potential anticancer properties against CRC cells in vitro. Nisin A induces pore formation on the target cell membrane with subsequent loss of plasma membrane integrity [189] and calcium influx, thus causing cell death [190]. Nisin Z presents selective toxicity against colon cancer HT-29 cells [191]. However, nisin Z failed to affect Caco-2 cells’ membrane integrity, and this discrepancy has been attributed to the purity of the nisin samples as well as to the nisin variant employed in each study, pointing out the need to take both factors under consideration in future studies [192]. Regarding nisin’s mode of action, it induces the intrinsic apoptotic pathway in CRC cells as indicated by *CASP3* and *CASP9* gene up-regulation [193] as well as the increased apoptotic index (Bax/Bcl-2 ratio) in two different studies [194,195]. Additionally, nisin down-regulates the expression of metastasis-related genes such as *MMPs*, carcinoembryonic antigen (*CEA*), and carcinoembryonic cell adhesion molecule 6 (*CEAM6*) [196]. Following nisin treatment, decreased expression of the cyclin D1 gene in CRC cells was observed, thus unveiling its crucial role in CRC progression [195]. Information pertaining to nisin’s in vivo antitumor effects is only limited to xenograft mouse models with head and neck squamous cell carcinoma (HNSCC) [190,197].

**Figure 2:**
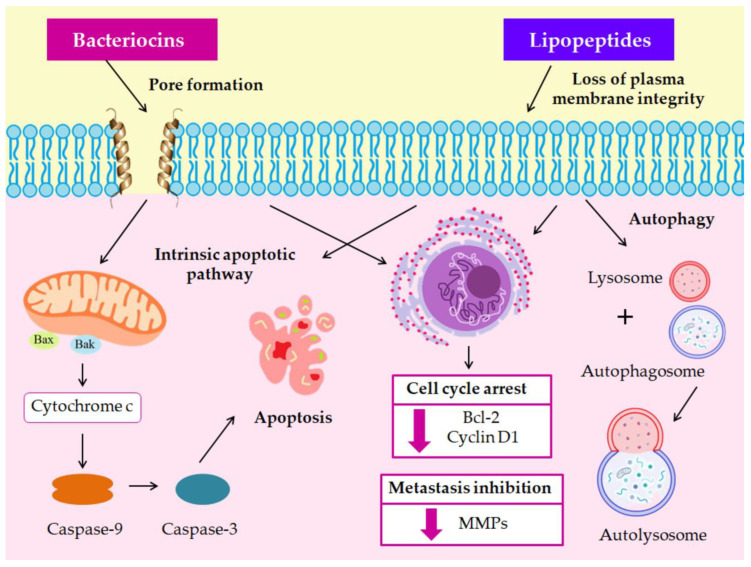
Cytotoxic effects of bacteriocins and nonribosomal bacterial lipopeptides.

A plethora of other bacteriocins exert antiproliferative activity against CRC cells, with a negligible effect against non-cancerous cells [198,199,200]. The in vitro cytotoxic effects of bacteriocins on CRC cells are shown in Figure 2 and summarized in Table 3. A recent meta-analysis study provided insight into the intervention of bacteriocins in various signaling cascades. For instance, they activate apoptosis via the regulation of the PI3K/AKT pathway, while they directly inhibit cyclooxygenase-2 (COX-2) expression and down-regulate the inflammatory NOD-like receptor family pyrin domain containing 3 (NLRP3) and nuclear factor-κB (NF-κB) pathways to diminish CRC-related inflammation [201]. Pediocin PA-1 induces cytotoxicity in HT-29 cells [202], possibly via interaction with TLRs based on 3D modeling approaches [203]. Plantaricin BM-1 triggers the caspase-dependent apoptotic pathway [204], while plantaricin P1053 increases the viability of normal CCD 841 cells via the activation of the epidermal growth factor receptor (EGFR) pathway [205]. Enterocin-treated cancer cells display apoptosis-like morphological changes [200,206].

### 3.4. Nonribosomal Lipopeptides

Nonribosomal lipopeptides derived from *Bacillus subtilis* are secondary bioactive molecules, synthesized by enzyme complexes, namely nonribosomal peptide synthetases (NRPS) [207]. These lipopeptides, mainly surfactin, iturin, and fengycin, exhibit significant cytotoxic activity against various CRC cell lines, thus contributing to elimination of cancer progression and metastasis [208] (Figure 2, Table 4). The underlying mechanisms involved in surfactin’s anticancer properties have recently been reviewed [209]. Surfactin can forcibly suppress CRC cell proliferation [210] via the induction of the caspase-dependent apoptotic pathway and cell cycle arrest at a certain concentration [211]. However, the major impediment to surfactin utilization as an anticancer agent is its hemolytic activity, leading to red blood cell (RBC) rupture and hemoglobin dissemination into the blood [211]. Iturin A can efficiently induce cytotoxic effects against CRC cells via multiple pathways, including initiation of paraptosis, apoptosis induction through the mitochondrial-mediated pathway, or activation of autophagy process [212]. Upon fengycin treatment in HT-29 cells, the expression of *BAX*, *CASP3*, and *CASP6* genes increases, while decreased levels of Bcl-2 protein are observed, indicating that the mitochondrial pathway of apoptosis is triggered [213].

### 3.5. Other Bacterial Peptides

Miscellaneous bacterial peptides of probiotic origin have emerged as novel promising treatment strategies for CRC (Table 4) [214,215]. Enterococcal antiproliferative peptide (Entap) demonstrates cytotoxic activity against HT-29 cells via the induction of apoptosis and cell cycle arrest in the G1 phase [216]. Mixirins, cyclic acyl-peptides derived from the marine bacterium *Bacillus* sp., can inhibit the proliferation of human HCT 116 cells [217]. AMPs and bacterial-derived protein-based therapeutics for tackling increasing CRC morbidity rates have been well documented [218,219]. For instance, LHH1, a novel AMP produced by *Lactobacillus casei* HZ1, increases CRC cell membrane susceptibility, causing irreversible damages [220]. Two other peptides, namely m2163 and m2386, can penetrate the cell cytoplasm to induce apoptosis in SW-480 cancer cells [221]. Additionally, KL15, the conjugated form of m2163 and m2386 peptides resulting from in silico modifications in their sequences, not only possesses potent antimicrobial activity against pathogens but also induces necrotic cell death [222]. Probiotic-derived ferrichrome acts as a tumor-suppressive molecule via the c-Jun N-terminal kinase (JNK) signaling pathway against cancerous IECs to a greater extent than conventional chemotherapeutic drugs, including cisplatin and 5-FU [223]. The probiotic-derived P8 protein was found to eliminate metastasis [224] and suppress CRC growth via the inhibition of the Wnt signaling pathway [225], whereas mucin binding protein (MucBP) exhibits dose-dependent antiproliferative effects against HT-29 cells [226].

**Table 4:** In vitro effects of nonribosomal and other bacterial peptides on CRC cells.

Class	Bioactive Compound	CRC Cell Line	Effect/Mode of Action	Reference
Nonribosomal peptides	Surfactin (*Bacillus subtilis*)	LoVo	dose- and time-dependent cytotoxic activity, caspase-dependent apoptosis induction, ERK and PI3K/AKT pathways suppression, cell cycle arrest (G0/G1)	[211]
		HCT-15, HT-29	dose-dependent cytotoxic activity	[210]
	Iturin A (*Bacillus subtilis*)	Caco-2	antitumor activity via multiple pathways: 1. intrinsic apoptotic pathway (↑ Bax, Bad/↓ Bcl-2), 2. paraptosis induction (ER dilatation, ↑ ROS production, ↑ Ca^2+^ levels, mitochondrial dysfunction), 3. autophagy (↑ LC3-II/↓ LC3-I)	[212]
	Fengycin (*Bacillus subtilis*)	HCT-15, HT-29	dose-dependent cytotoxic activity	[210]
		HT-29	↓ cell proliferation, cell cycle arrest (G1), apoptosis induction, ↑ ROS production, ↑ Bax and caspase-3, -6/↓ Bcl-2 and CDK4/cyclin D1	[213]
Other bacterial peptides	Entap (*Enterococcus* sp.)	HT-29	apoptosis induction, cell cycle arrest (G1)	[216]
	Ferrichrome (*Lactobacillus casei* ATCC 334)	Caco-2, SW-620, SK-CO-1	tumor-suppressive effect, apoptosis induction via inhibition of JNK pathway	[223]
	KL15 peptide (*Lactobacillus casei* ATCC 334)	SW-480, Caco-2	antiproliferative effect, increased membrane permeability, necrotic cell death	[222]
	LHH1 peptide (*Lactobacillus casei* HZ1)	HCT 116	dose-dependent cytotoxic effect, apoptosis induction, membrane damage	[220]
	m2163 and m2386 peptides (*Lactobacillus casei* ATCC 334)	SW-480	↓ cell proliferation, extrinsic and intrinsic apoptosis induction, ↑ FasR and *TRAILR1* expression (m2163)/ ↑ FasR, TNFR1, and *TRAILR1* (m2386)	[221]
	Mixirins (*Bacillus* sp.)	HCT 116	↓ cell proliferation	[217]
	MucBP (*Lactobacillus casei*)	HT-29	dose-dependent antiproliferative effect	[226]
	Probiotic-derived P8 protein (*Lactobacillus rhamnosus* KCTC 12202BP)	DLD-1	antiproliferative and anti-migration activity, cell cycle arrest (G2), p53-p21-Cyclin B1/CDK1 pathway inhibition	[224]
			Wnt pathway suppression (dysregulation of GSK3β transcription), cell cycle arrest	[225]

↑: increase or up-regulation, ↓: decrease or down-regulation. Colon cancer cell lines: LoVo, HCT-15, HT-29, Caco-2, SW-620, SK-CO-1, SW-480, HCT 116, DLD-1. ER: Endoplasmic reticulum, ROS: Reactive oxygen species, LC3-II: Microtubule-associated protein 1A/1B-light chain 3-II, LC3-I: Microtubule-associated protein 1A/1B-light chain 3-I, CDK4: Cyclin-dependent kinase 4, JNK: c-jun N-terminal kinase, FasR: Fas receptor, *TRAILR1:* TRAIL receptor 1 gene, TNFR1: Tumor necrosis factor receptor-1, MucBP: Mucin binding protein, CDK1: Cyclin-dependent kinase 1, GSK3β: Glycogen synthase kinase β.

### 3.6. Short-Chain Fatty Acids

Short-chain fatty acids (SCFAs) constitute a group of metabolic products originating from the microbial fermentation of non-digestible carbohydrates [227]. The intestinal epithelium absorbs almost 95% of SCFAs synthesized by the gut microbiota [228]. SCFAs contribute to the maintenance of homeostasis, enhance gut barrier integrity, and participate in the energetic metabolism [229]. Upon their production, SCFAs are transported into the IECs via the SMCT1 [230]. Apart from the intestinal environment, a small amount of SCFAs that are not metabolized by colonocytes can reach systemic circulation and disseminate to distant tissues and organs [229], acting as signaling molecules with profound health benefits to the host [231].

**Figure 3:**
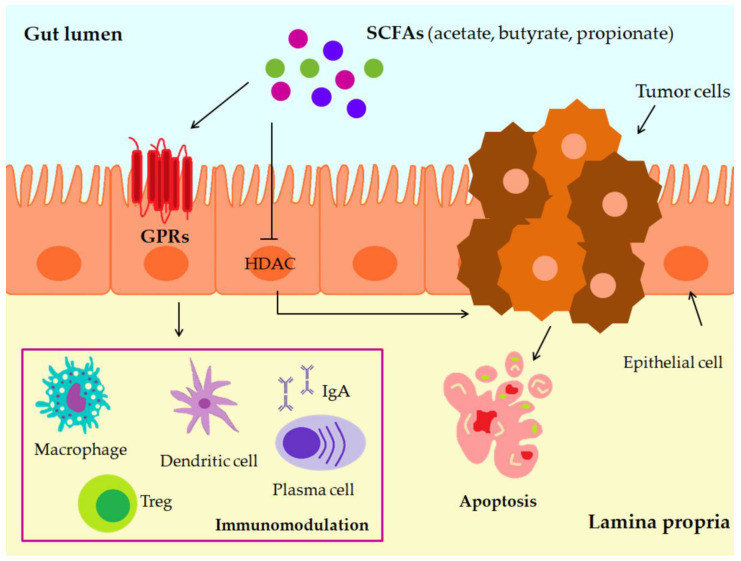
SCFAs effects on CRC cells: histone deacetylases (HDACs) inhibition and cell surface G-protein-coupled receptors (GPR) activation, immunomodulation, apoptosis.

SCFAs effects are mediated by two main pathways: the inhibition of histone deacetylases (HDACs) and the activation of cell surface G-protein-coupled receptors (GPRs), namely GPR41, GPR43, and GPR109A [232] (Figure 3). The administration of a mix of SCFAs (acetate, butyrate, and propionate) in a mouse model of colitis-associated CRC significantly reduced tumor incidence and attenuated colonic inflammation [233]. GPR43 deficiency was found to promote the progression of adenoma to adenocarcinoma in vivo [234]. SCFA administration suppressed intestinal inflammation and carcinogenesis in GPR43-deficient mice [235].

The pivotal role of SCFAs in the elimination of colorectal malignancy has already been reviewed [236,237,238]. A recent meta-analysis study demonstrated that lower fecal concentrations of the major SCFAs are correlated with increased CRC incidence [239]. The prominent mechanisms of SCFA action involve the down-regulation of genes related to DNA replication [240], the promotion of cell-cycle arrest and apoptosis [241], and the regulation of complex immune responses [242,243]. SCFAs provide resistance toward enteric bacterial pathogens associated with CRC development and progression [244].

Among the three aforementioned SCFAs, the anticancer effects of butyrate on CRC cells are most well-documented [245,246,247]. Butyrate can inhibit CRC proliferation via multiple mechanisms, such as the induction of the autophagy-mediated degradation of β-catenin [248], epigenetic reprogramming [249], the up-regulation of TLR4 expression, and the activation of the mitogen-activated protein kinase (MAPK) and NF-κΒ pathways [250]. Furthermore, it induces CRC cell ferroptosis via the CD44/Solute Carrier Family 7 Member 11 (SLC7A11) pathway and exhibits a synergistic therapeutic effect when combined with erastin, a ferroptosis-positive drug [251]. Interestingly, butyrate restores cytokine-induced barrier disruption, contributing to the maintenance of intestinal homeostasis [252].

Acetate was shown to reduce CRC proliferation and induce apoptosis, as indicated by several features such as loss of mitochondrial membrane potential, nuclear chromatin condensation, and ROS generation [253]. Upon acetate treatment, CRC cells exhibit apoptosis-related morphological features, while lysosomal membrane permeabilization with subsequent cathepsin D release in the cytosol takes place [254]. Another study suggests that acetate’s antiproliferative effect is a consequence of its impact on mitochondrial metabolism [255]. Propionate was found to down-regulate the protein arginine methyltransferase 1 (PRMT1) and regulate the mTOR pathway in HCT 116 cells [256]. Furthermore, it can suppress CRC tumorigenesis through promoting the proteasomal degradation of euchromatic histone-lysine N-methyltransferase 2 (EHMT2) through HECT domain E3 ubiquitin protein ligase 2 (HECTD2) up-regulation [257].

## 4. Challenges and Future Perspectives

Accumulating evidence has elucidated that gut microbiota dysbiosis contributes perilously to CRC occurrence and progression. Detrimental opportunistic pathogens can reconstruct the composition of colonic commensal bacteria, thus favoring the creation of a microenvironment susceptible to carcinogenesis. Conventional CRC treatments are characterized by the insufficient ability to specifically target cancer cells, while they are accompanied by chemoresistance development and numerous side effects in the host. Probiotic supplementation has been recommended as an effective complementary therapy for the elimination of gastrointestinal discomfort and the attenuation of gut dysbiosis in CRC patients undergoing chemotherapy [18]. However, despite probiotics’ accredited beneficial health effects, including epithelial colonization, the restoration of microbial diversity, and the detoxification of carcinogens, a few studies have highlighted impediments to their utilization [20]. In that context, miscellaneous bioactive compounds of probiotic origin with antiproliferative properties, such as bacteriocins, SCFAs, and EPSs could be exploited as conceivable anticancer agents [258]. Bacteriocins have been predominantly used as natural biopreservatives in the food industry, with nisin being the only bacteriocin licensed by the Food and Drug Administration (FDA) as a “GRAS” additive [259]. Apart from food applications, bacteriocins are now receiving increased attention as promising anticancer agents due to their specificity against cancer cells with limited or no effect on healthy cells [178]. Nevertheless, despite their advantageous properties of relevance to medical use, including biocompatibility, biodegradability, and lack of immunogenicity [180], they display some fundamental shortcomings, such as decreased bioavailability and susceptibility to proteolytic enzymes during exposure in the GIT, when orally administrated [259]. To overcome such limitations, multiple strategies varying from encapsulation technologies to bioengineering and semi-synthetic techniques could be recruited to improve their physicochemical characteristics and biological activity, while high-throughput sequencing may enhance the discovery of new bacteriocins [260]. Additionally, the costly production pertaining to bacteriocins’ efficient purification [261] as well as their high complexity due to extended posttranslational modifications [260] remain important hindrances towards their large-scale manufacturing. Interestingly, as progressive increases in multidrug-resistant infections have been declared a global health emergency [262], bacteriocins are considered as next-generation antibiotics [259], as well as feasible microbiome- editing tools [263] due to their potent antimicrobial activity. In fact, bacteriocins’ antimicrobial and toxic effects, as well as their biosafety in in vivo systems have been recently addressed [262,264]. They were found to act as immunostimulatory molecules contributing to the reduction of infection-associated parameters, including biochemical and histopathological biomarkers [262]. Furthermore, the anticancer effects of microcin E492 were tested in zebrafish xenografts, showing significant reduction in tumor growth [265], while nisin was found to act synergistically with the chemotherapeutic 5-FU in murine skin cancer models [266]. However, there is still inadequate research regarding their toxicity and therapeutic efficacy in vivo, a crucial prerequisite towards clinical trials. Indeed, in vitro experiments may not necessarily align with in vivo studies. Hence, further investigations should be conducted to decipher the delivery strategies, route of administration, and pharmacokinetic parameters of bacteriocins.

EPSs are complex, multifunctional carbohydrates excreted from probiotic bacteria, which have recently gained research attention with regard to pharmaceutical and therapeutic applications [150] due to their favorable health-promoting properties, such as immune system modulation, free radicals scavenging, and inhibition of cancer cell growth [267]. They are considered promising substitutes for synthetic anticancer drugs owing to their unique features, including biocompatibility, thermal stability, biodegradability, and nontoxic nature. The diverse physicochemical characteristics of EPSs (e.g., monosaccharide composition, branching degree, electric charge, molecular weight) are directly associated with their functionality [268]. Therefore, specific methodologies should be implemented to elucidate their chemical structure and provide an insight on their structure-dependent functional benefits [267,268,269,270]. Additionally, the high production costs and low polysaccharide yields as well as the time-consuming processes required for EPS purification currently restrict their commercialization [269]. Regarding this aspect, genetic and metabolic engineering could facilitate EPS production yield [268]. Detailed studies should also be performed to evaluate their safety via the application of targeted high-throughput screening strategies [271]. Only limited research has been performed in vivo, supporting probiotic EPSs’ ability to alleviate intestinal inflammation via gut microbiota modulation [272,273,274], contribute to the maintenance of the epithelial barrier [275], exert anticancer effects [276,277], and attenuate 5-FU-induced toxicity in animal models [278]. Nevertheless, there is still a lack of clinical evidence to proceed into human administration.

SCFAs represent the largest group of bioactive compounds residing in the intestinal lumen with a profound contribution to gut immunity stimulation. SCFAs also act as signaling molecules upon their dissemination into the bloodstream, thus promoting health benefits for the host [37]. Given their protective anti-inflammatory and antioxidant features [279], SCFAs have been found to eliminate the severity of conventional anticancer drugs’ GIT toxicities [280]. They can also exert anticancer properties via the regulation of immune response, a reduction in HDAC activity [281], and the repairment of intestinal microecology [282], thus promoting chemosensitivity and cell growth inhibition, or they can act as antitumor adjuvant drugs [283]. Patients responding to chemotherapy, immunotherapy, and radiotherapy treatments were found to exhibit a higher abundance of SCFA-producing microbes and higher levels of fecal and plasma SCFAs. Furthermore, recent studies suggest that SCFA-based interventional strategies could be implemented to promote cancer treatment efficacy and decrease the adverse side effects commonly caused by chemotherapeutics [284,285]. However, the majority of them focus on defining the correlation between SCFAs levels and therapeutic outcomes rather than assessing SCFAs’ utilization for medical purposes. Inconsistent results derived from in vitro and in vivo studies may be attributed to different methodological approaches as well as interindividual variations in SCFA production [284]. These limitations should be taken under consideration prior to experimental designs. Hence, the lack of pre-clinical and clinical evidence still impedes the evaluation of SCFAs’ local and systemic effects and the determination of the favorable route of administration to the host. In this context, more elaborative investigations are required to extrapolate conclusions regarding the multifaceted interactions occurring in the gut and configure the criteria of SCFA utilization based on the distinctiveness of each individual.

## Figures and Tables

**Table 2:** In vitro effects of exopolysaccharides on CRC cells.

Probiotic Strain	CRC Cell Line	Effect/Mode of Action	Reference
*Lactobacillus* spp.			
*L. acidophilus* 10307	Caco-2	dose-dependent anticancer activity (in both normoxic and hypoxic conditions), ↑ *PPARG*, ↑ *EPO* under normoxia	[172]
	HCT-15, Caco-2	↓ cell proliferation, reduction of membrane integrity, antioxidative properties (↑ *HMOX1*), ↓ *VEGF* and *HIF1A*, ↑ *TIMP3* and *HIF2A*, ↑ PAI-1 gene	[171]
*L. acidophilus* 606	HT-29	activation of autophagic cell death via Beclin-1, GRP78, and Bak induction	[173]
*L. acidophilus* DSMZ 20079	Caco-2	↓ cell proliferation, cell cycle arrest (G0/G1), morphological changes related to apoptosis (shrinkage, membrane blebbing), NF-κB inflammatory pathway inactivation	[168]
*L. brevis* LB63	HT-29	time-dependent antiproliferative effect, apoptosis induction (↑ Bax, caspase-3, -9/↓ Bcl-2 and survivin)	[163]
*L. brevis* TD4	HT-29	dose and time-dependent cytotoxic activity, apoptosis induction (↑ DNA fragmentation)	[169]
*L. casei* 01	HT-29	dose-dependent antiproliferative effect, reduction of pro-mutagen’s 4-NQO cytotoxicity	[174]
*L. casei* SB27	HT-29	↓ cell proliferation, apoptotic morphological changes, ↑ *BAD*, *BAX*, *CASP3*, *CASP8*	[161]
*L. casei* strains (K11, M5, SB27, and X12)	HT-29	dose-dependent antiproliferative effects, cell cycle arrest (G0/G1), apoptotic bodies formation, ↑ caspase-3	[167]
*L. delbrueckii* ssp. *bulgaricus* B3	HT-29	time-dependent antiproliferative effect, apoptosis induction (↑ Bax, caspase-3, - 9/↓ Bcl-2 and survivin)	[163]
*L. delbrueckii* ssp. *bulgaricus* DSM 20080	Caco-2	antioxidative and antitumor properties, apoptosis induction (↑ *BAX*, *CASP3*, *CASP8*, *p53*/ ↓ *BCL2*, *MCL1*, *Vimentin*)	[165]
*L. fermentum* YL-11	HT-29, Caco-2	dose-dependent antitumor effect, nuclear condensation related to apoptosis	[170]
*L. helveticus* MB2-1	Caco-2	dose and time-dependent anticancer effect	[155]
*L. kefiri* MSR101	HT-29	dose-dependent anticancer activity, apoptosis induction (↑ cyt c, Bax, Bad, and caspase-3, -8, -9)	[162]
*L. paracasei* TD3	HT-29	dose and time-dependent cytotoxic activity, apoptosis induction (↑ DNA fragmentation)	[169]
*L. plantarum*-12	HT-29	↓ cell proliferation, ↑ ROS production, intrinsic apoptotic pathway (↑ Bax, caspase-3, -8, -9/↓ Bcl-2), PCNA inhibition in dose-dependent manner	[164]
*L. plantarum* 70810	HT-29	dose and time-dependent antitumor effect	[154]
*L. plantarum* GD2	HT-29	time-dependent antiproliferative effect, apoptosis induction (↑ Bax, caspase-3, -9/↓ Bcl-2 and survivin)	[163]
*L. plantarum* NCU116	CT26	↓ cell proliferation, ↑ TLR2, c-Jun dependent Fas/FasL-mediated apoptotic pathway	[166]
*L. plantarum* NRRL B- 4496	HCT 116, Caco-2	dose-dependent antitumor activity	[153]
*L. plantarum* WLPL04	HT-29	dose and time-dependent antitumor effect, inhibition of *E. coli* adhesion to HT-29 cells	[157]
*L. plantarum* YW32	HT-29	dose and time-dependent anticancer activity	[156]
*L. rhamnosus* E9	HT-29	time-dependent antiproliferative effect, apoptosis induction (↑ Bax, caspase-3, -9/↓ Bcl-2 and survivin)	[163]
Others			
*Lactococcus garvieae* C47	Caco-2	antioxidant and antitumor activity	[158]
*Pediococcus acidilactici* NCDC 252	HCT 116	dose-dependent antiproliferative activity	[160]
*Pediococcus pentosaceus* M41	Caco-2	antioxidant and antitumor activity	[159]
Yeasts			
*Kluyveromyces marxianus*, *Pichia kudriavzevii*	SW-480, HT-29, HCT 116	↓ cell proliferation, suppression of AKT-1, JAK-1 and mTOR pathways, apoptosis induction (↓ *BCL2*/↑ *BAX*, *CASP3*, *CASP8*)	[175]

↑: increase or up-regulation, ↓: decrease or down-regulation. Colon cancer cell lines: Caco-2, HCT-15, HT-29, CT26 (mouse epithelial colorectal cell line), HCT 116, SW-480. *PPARG*: Peroxisome proliferator-activated receptor-gamma gene, *EPO*: Erythropoietin gene, *HMOX1*: Hemeoxygenase-1 gene, *VEGF*: Vascular endothelial growth factor gene, *HIF*: Hypoxia-inducible factor gene, *TIMP3*: Tissue inhibitor of metalloproteinase-3 gene, PAI-1: Plasminogen activator inhibitor-1, GRP78: G-protein coupled receptor, NF-κB: Nuclear factor-κB, 4-NQO: 4-nitroquinoline 1-oxide, *CASP*: caspase gene, *MCL1*: Myeloid leukemia 1 gene, cyt c: cytochrome c, ROS: Reactive oxygen species, PCNA: Proliferating cell nuclear antigen, TLR2: Toll-like receptor 2, c-Jun: transcription factor, FasL: Fas ligand, AKT-1: AKT serine/threonine kinase 1, JAK-1: Janus kinase, mTOR: mammalian target of rapamycin.

**Table 3:** In vitro effects of bacteriocins on CRC cells.

Bacteriocin	CRC Cell Line	Effect/Mode of Action	Reference
Duramycin (*Streptomyces* sp.)	Caco-2, HCT 116, LoVo	detection of PE on cell surface, dose- and time-dependent Ca^2+^ release	[185]
Enterocin 12a (*Enterococcus faecium* 12a)	HCT-15	dose-dependent antiproliferative activity, morphological changes related to apoptosis	[200]
Enterocin-A (*Enterococcus faecium* por1)	HT-29, Caco-2	dose-dependent cytotoxic effect, morphological changes related to apoptosis, cell cycle arrest (G1)	[198]
Heterodimer Enterocin-A + B (*Enterococcus faecium*)	HT-29	improved cytotoxicity compared to enterocin-B alone, apoptosis related morphological changes	[199]
Enterocin OE-342 (*Enterococcus faecalis* OE-342)	HCT 116	dose-dependent cytotoxic effect, immunomodulatory activity, cell cycle arrest (G2/M), morphological changes related to apoptosis	[206]
Nisin A (*Lactococcus lactis* subsp. *lactis*)	Caco-2, HT-29	↓ cell proliferation, loss of plasma membrane integrity	[189]
	SW-480	dose-dependent cytotoxic effect, intrinsic apoptotic pathway (↑ Bax/Bcl-2 ratio)	[194]
	LS 180, HT-29, SW48, Caco-2	↓ cell proliferation, anti-metastatic effects (↓*CEA*, *CEAM6*, *MMP2F*, and *MMP9F*)	[196]
	SW-480	dose-dependent cytotoxic effect, ↑ *BAX*/*BCL2* ratio, ↑*CASP3*, *CASP9*	[193]
		dose-dependent cytotoxic effect, ↓ *CCND1*	[195]
Pediocin PA-1 (*Pediococcus acidilactici* K2a2-3)	HT-29	↓ cell proliferation	[202]
Plantaricin BM-1 (*Lactobacillus plantarum* BM-1)	SW-480, Caco-2, HCT 116	dose-dependent cytotoxic effect, morphological changes related to apoptosis, caspase-dependent apoptosis pathway (PARP-1 cleavage, dysregulation of TNF, NF-κB, and MAPK signaling pathways)	[204]
Plantaricin P1053 (*Lactobacillus plantarum* PBS067)	E705	dose-dependent cytotoxic effect	[205]

↑: increase or up-regulation, ↓: decrease or down-regulation. Colon cancer cell lines: Caco-2, HCT 116, LoVo, HCT-15, HT-29, SW-480, LS 180, SW48, E705. PE: Phosphatidylethanolamine, *CEA*: Carcinoembryonic antigen gene, *CEAM6*: Carcinoembryonic cell adhesion molecule 6 gene, *MMP2F*: Matrix metalloproteinase-2F gene, *MMP9F*: Matrix metalloproteinase-9F gene, *CASP*: caspase gene, *CCND1*: cyclin D1 gene, PARP-1: Poly (ADP-ribose) polymerase-1, TNF: Tumor necrosis factor, NF-κB: Nuclear factor-κB, MAPK: Mitogen-activated protein kinase.

## Data Availability

Not applicable.
